# COX-2 expression positively correlates with PD-L1 expression in human melanoma cells

**DOI:** 10.1186/s12967-017-1150-7

**Published:** 2017-02-23

**Authors:** Gerardo Botti, Federica Fratangelo, Margherita Cerrone, Giuseppina Liguori, Monica Cantile, Anna Maria Anniciello, Stefania Scala, Crescenzo D’Alterio, Chiara Trimarco, Angela Ianaro, Giuseppe Cirino, Corrado Caracò, Maria Colombino, Giuseppe Palmieri, Stefano Pepe, Paolo Antonio Ascierto, Francesco Sabbatino, Giosuè Scognamiglio

**Affiliations:** 1 Dipartimento di Patologia Diagnostica e di Laboratorio: SC di Anatomia Patologica e Citopatologia, Istituto Nazionale Tumori IRCCS Fondazione “G. Pascale”, Via Mariano Semmola, 80131 Naples, Italy; 2 Struttura Complessa di Oncologia Medica e Terapie Innovative, Istituto Nazionale Tumori IRCCS Fondazione “G. Pascale”, Via Mariano Semmola, 80131 Naples, Italy; 3 Genomica Funzionale, Istituto Nazionale Tumori IRCCS Fondazione “G. Pascale”, Via Mariano Semmola, 80131 Naples, Italy; 4 Department of Pharmacy, University of Naples “Federico II”, 80131 Naples, Italy; 5 Melanoma and Sarcoma Surgery Unit, Istituto Nazionale Tumori IRCCS Fondazione “G. Pascale”, Via Mariano Semmola, 80131 Naples, Italy; 6 Unit of Cancer Genetics, Institute of Biomolecular Chemistry, National Research Council, 07100 Sassari, Italy; 7 Department of Medicine and Surgery, University of Salerno, Baronissi, 84081 Salerno, Italy

**Keywords:** Immune checkpoint molecules, PD-L1, COX-2, Celecoxib, Melanoma, Immunotherapy

## Abstract

**Background:**

The resistance to PD-1/PD-L1 inhibitors for the treatment of melanoma have prompted investigators to implement novel clinical trials which combine immunotherapy with different treatment modalities. Moreover is also important to investigate the mechanisms which regulate the dynamic expression of PD-L1 on tumor cells and PD-1 on T cells in order to identify predictive biomarkers of response. COX-2 is currently investigated as a major player of tumor progression in several type of malignancies including melanoma. In the present study we investigated the potential relationship between COX-2 and PD-L1 expression in melanoma.

**Methods:**

Tumor samples obtained from primary melanoma lesions and not matched lymph node metastases were analyzed for both PD-L1 and COX-2 expression by IHC analysis. Status of BRAF and NRAS mutations was analyzed by sequencing and PCR. Co-localization of PD-L1 and COX-2 expression was analyzed by double fluorescence staining. Lastly the BRAF^V600E^ A375 and NRAS^Q61R^ SK-MEL-2 melanoma cell lines were used to evaluate the effect of COX-2 inhibition by celecoxib on expression of PD-L1 in vitro.

**Results:**

BRAF^V600E/V600K^ and NRAS^Q61R/Q61L^ were detected in 57.8 and 8.9% of the metastatic lesions, and in 65.9 and 6.8% of the primary tumors, respectively. PD-L1 and COX-2 expression were heterogeneously expressed in both primary melanoma lesions and not matched lymph node metastases. A significantly lower number of PD-L1 negative lesions was found in primary tumors as compared to not matched metastatic lesions (*P* = 0.002). COX-2 expression significantly correlated with PD-L1 expression in both primary (*P* = 0.001) and not matched metastatic (*P* = 0.048) lesions. Furthermore, in melanoma tumors, cancer cells expressing a higher levels of COX-2 also co-expressed a higher level of PD-L1. Lastly, inhibition of COX-2 activity by celecoxib down-regulated the expression of PD-L1 in both BRAF^V600E^ A375 and NRAS^Q61R^ SK-MEL-2 melanoma cell lines.

**Conclusions:**

COX-2 expression correlates with and modulates PD-L1 expression in melanoma cells. These findings have clinical relevance since they provide a rationale to implement novel clinical trials to test COX-2 inhibition as a potential treatment to prevent melanoma progression and immune evasion as well as to enhance the anti-tumor activity of PD-1/PD-L1 based immunotherapy for the treatment of melanoma patients with or without BRAF/NRAS mutations.

**Electronic supplementary material:**

The online version of this article (doi:10.1186/s12967-017-1150-7) contains supplementary material, which is available to authorized users.

## Background

The recent introduction of new and more effective therapies, including treatments based on the stimulation of immune response and targeted therapies, has partially changed the prognosis of metastatic melanoma patients [1]. The more effective therapeutic strategies are based on tyrosine kinase inhibitors, such as vemurafenib, dabrafenib and trametinib or inhibitors of the immune checkpoint molecules Cytotoxic T lymphocyte antigen 4 (CTLA-4), Programmed death receptor 1 (PD-1) and its ligand PD-L1 [2–6].

The immune system plays an important role in eradicating melanoma cells. PD-1 is a T cell co-inhibitory receptor with ligand specificity for both PD-L1 and PD-L2. PD-L1 results to be expressed in various types of cells, including placenta, pancreatic islet cells, mesenchymal stem cells and immune cells. However many human cancers, including melanoma, breast, lung, stomach, pancreatic, kidney and ovarian carcinoma are shown to express PD-L1 and in melanoma, its expression correlates with a poor prognosis [7–12]. Interaction of PD-1 with PD-L1 (B7-H1) or PD-L2 (B7-DC) represents one of the major mechanisms of tumor immune escape [13]. It promotes T-cell tolerance and avoids T cell cytolysis of cancer cells.

Several mechanisms have been implicated in the regulation of PD-L1 expression by cancer cells such as activation of mitogenic and pro-survival pathways including MAPK and PI3K/AKT, increased activity of transcriptional factors HIF-1, STAT-3 and NF-κB, and presence of epigenetic modulators including miR-513, miR-570, miR-34a, miR-200 and miR-197 [14].

Cyclooxygenases (COXs) are enzymes which catalyze the first rate-limiting step in the conversion of arachidonic acid to prostaglandins. Two COX isoenzymes have been identified: COX-1 is constitutively expressed in most tissues and mediates the synthesis of prostaglandins in normal physiological processes, whereas COX-2 is not detectable in most normal tissues but is rapidly induced by various stimuli such as inflammatory reactions [15]. COX-2 is also expressed in various tumor types and its level correlates with invasiveness and prognosis in many tumor entities, suggesting an important role of COX-2 in tumor development and progression [16]. Epidemiological studies showed that prolonged COX-2 inhibition through acetylsalicylic acid or other non-steroidal anti-inflammatory drugs (NSAIDs) might offer some protection against colon cancer and some other malignancies [17–19]. Moreover, a potential role of COX-2 in melanoma development is also not unlikely, since COX-2 is frequently expressed in malignant melanomas [20] and its inhibition may prevent melanoma progression [21]. Evidences available in the literature have shown that COX-2 can modulate PD-L1 expression in breast cancer cells [22] and its inhibition by the selective COX-2 inhibitor celecoxib enhances the effects of cytotoxic T lymphocyte (CTL) function by PD-L1 blocking in chronic viral infections [23].

In this study we tested whether COX-2 expression correlates with PD-L1 expression in primary and not matched metastatic melanoma tumors as well as whether COX-2 activity regulates PD-L1 expression in melanoma cells.

## Methods

### Chemical reagents and antibodies

COX-2-specific mouse monoclonal antibody (mAb) (clone CX-294), mouse IgG2a, rabbit IgG, the peroxidase blocking reagent goat anti-rabbit + horseradish peroxidase (HRP) visualization reagent, DAB substrate buffer, DAB chromogen, bond wash solution, hematoxylin and EnVision FLEX + rabbit linker kitwere purchased from DAKO. PD-L1-specific rabbit mAb (clone SP-142) was purchased from Spring Bioscience. Cell Conditioning Solution was purchased from Ventana medical Systems. Protein serum block, steady plus 3,3′-diaminobenzidine (DAB) kits, goat anti-mouse IgG dylight 488 and goat anti-rabbit IgG dylight 594 were purchased from Abcam. Bond primary antibody diluents and 4′,6′-diamidino-2-phenylindole (DAPI) were purchased from Leica biosystems. MACH 2 DOUBLE STAIN 2 and vulcan fast red chromogen Kit 2 were purchased from Biocare. The COX-2 inhibitor, celecoxib was purchased from Selleck Chemicals LLC. 3-(4,5-dimethylthiazol-2-yl)-2,5-diphenyltetrazoliumbromide (MTT) and trypan blue were purchased from Sigma. GAPDH-, Bcl-2- and β-actin specific mAbs were purchased from Cell Signaling Technology. R-phycoerythrin(PE)-conjugated PD-L1-specific mouse mAb [clone MIH1 (RUO)] and PE-conjugated mouse IgG1 were purchased from BD Biosciences.

### Tumor samples

Primary melanoma tumor biopsies from treatment naïve patients and not matched lymph node melanoma tumor biopsies from metastatic patients were obtained from the tissue bank at Istituto Nazionale Tumori Fondazione “G. Pascale” (Naples, Italy). All samples were from Caucasian patients. Patients were consented for tissue acquisition per institutional review board (IRB)-approved protocol. Presence of tumor cells in formalin-fixed paraffin embedded (FFPE) tissues was monitored by hematoxylin and eosin (H&E) staining. All samples were reviewed by two experienced pathologists (GB and AA) according to American Joint Committee on Cancer (AJCC) classification criteria, using standard tissue sections and appropriate immunohistochemical (IHC) analyses.

### Genotyping of metastatic lymph node melanoma tumors

Genomic DNA was isolated from FFPE tumor tissues, using the QIAamp DNA FFPE tissue kit (QIAGEN Inc; Milan, Italy). The full coding sequences and splice junctions of *NRAS* (exons 1 and 2) and the entire sequence of the *BRAF* (exon 15) [24, 25] were screened for mutations. All samples were assessed for the quality of the purified DNA, in order to avoid that discrepant case could arise from insufficient sample quality. Primer sets were designed as described [26]. Sequencing and PCR assay were performed as described [26].

### IHC analysis

FFPE tumor tissue sections of 3–4 μm thickness were cut onto adesive slides, baked at 70 °C (dry heat) for 1 hour (h) less than 1 week before use, deparaffinized in four changes of 100% xylene, and rehydrated with a graded ethanol series (100, 70, 40%) to distilled water. COX-2 IHC staining was performed using COX-2-specific mAb (clone CX-294) and EnVision FLEX utilizing the automated DAKO Omnis platform. For PD-L1 staining, prepared slides were incubated for 12 minutes (min) at 110 °C in Cell Conditioning Solution, using a commercial steamer as the heat source (Biocare Medical, Decloaking Chamber DC12). After cooling for 20 min, PD-L1 staining was performed using an automated IHC staining platform (DAKO autostainer Link48). This procedure was carried out at room temperature (RT). Following a 5 min incubation with a peroxidase blocking reagent and a 5 min incubation with a protein serum block (1% goat serum, 4% BSA in PBS, slides were incubated with the PD-L1-specific mAb (clone SP-142) at a concentration of 3.75 μg/mL in the primary antibody diluents for 90 min. The goat anti-rabbit + HRP visualization reagent, which is biotin-independent and reduces the potential for background or nonspecific staining from endogenous biotin, was used for PD-L1-specific antibody detection. The secondary antibody was incubate for 40 min [27].

For both COX-2 and PD-L1 staining, following an incubation with DAB substrate buffer and DAB chromogen, slides were counterstained on platform with hematoxylin and rinsed in distilled water. Between all incubation steps, slides were extensively washed with bond wash solution. Then the slides were dehydrated out of platform in an ethanol series (30, 70, 100%) and four changes of 100% xylene, and permanently sealed with automatic coverslips (DAKO #CS100). Each staining run contained positive and negative controls.

PD-L1 and COX-2 expression were reviewed and enumerated independently and blindly by two experienced pathologists (GB and AA) using a light microscopy. For each sample, at least five fields (inside the tumor and in the peripheral areas) and >500 cells were analysed. Using a semi-quantitative scoring system microscopically and referring to each protein scoring method in other studies, percentage of stained tumor cells in each lesion were evaluated for COX-2 and PD-L1 expression. Variations in the percentage of stained cells were within a 10% range for COX-2 expression. In consideration of the error, we evaluated the percentage of stained tumor cells at 10% intervals. Staining was graded as a semi-quantitative variable ranged from 0 to 100% [28]. For PD-L1 expression variations in the percentage of stained cells were within a 1% range. Results were graded as negative (0+), light positive (1+) and positive (2+) when the PD-L1 score in an entire lesion was 0, 1–5, and >5% respectively [29, 30].

### Immunofluorescence staining

Double-fluorescence staining of PD-L1 and COX-2 were conducted on a total of 12 representative FFPE tissue sections, 6 each from primary and metastatic lesions, including both positive and negative for PD-L1 (clone SP-142) or COX-2 (clone CX-294) at IHC staining. Following their deparaffinization and hydration, prepared slides were incubated with antigen retrieval in a pressure cooker (Biocare) for 10 min at 110 °C. Following an incubation with protein blocking, slides were incubated with a cocktail of PD-L1- and COX-2-specific mAbs at RT. Primary antibodies were detected utilizing a cocktail of goat anti-mouse IgG dylight 488 and goat anti-rabbit IgG dylight 594. After washing for two times, nuclei were stained with DAPI at RT for 10 min, and stored at 4 °C. The slides were examined using a fluorescent microscope (Olympus BX61).

### Cell lines

The BRAF^V600E^ A375 and the NRAS^Q61R^ SK-MEL-2 melanoma cell lines were purchased from American Type Culture Collection (ATCC). All cell lines were cultured in RPMI 1640 medium (HyClone Laboratories) supplemented with 2 mmol/l l-glutamine (HyClone Laboratories) and 10% fetal bovine serum (FBS) (HyClone Laboratories). All cells were cultured at 37 °C in a 5% CO_2_ atmosphere.

### Cell proliferation and MTT assay

Cells were plated in triplicate in 96-well microtiter plates at the density of 3 × 10^3^ per well in 100 μl of RPMI 1640 medium supplemented with 10% FBS at 5% CO_2_ atmosphere and treated with celecoxib. Untreated cells were used as a control. Dimethyl sulfoxide (DMSO) (vehicle of the drug) concentration was maintained at 0.02% in all wells. Cell proliferation was evaluated at the indicated time point utilizing the MTT assay which was carried out as reported elsewhere [31]. Data are expressed as percent of proliferation of treated cells as compared to untreated control cells. All experiments were performed three independent times in triplicates. The absorbance value at wavelength of 540 nm was determined using a microplate reader (Bio-Rad Laboratories).

### Cell viability assay

Cells were seeded at the density of 4 × 10^5^ in a 75 cm^2^ tissue culture flask in RPMI 1640 medium supplemented with 10% FBS at 5% CO_2_ atmosphere and incubated with the indicated doses of celecoxib. Untreated cells were used as a control. The DMSO (vehicle of the drug) concentration was maintained at 0.02% in all flasks. Following a 24 h incubation at 37 °C in a 5% CO_2_ atmosphere, cell viability was evaluated utilizing the trypan blue assay which was carried out as reported elsewhere [32].

### Western blot analysis

Cells were seeded at the density of 4 × 10^5^ in a 75 cm^2^ tissue culture flask in RPMI 1640 medium supplemented with 10% FBS at 5% CO_2_ atmosphere and incubated with the indicated doses of celecoxib. Untreated cells were used as a control. The DMSO (vehicle of the drug) concentration was maintained at 0.02% in all flasks. Following a 24 h incubation at 37 °C in a 5% CO_2_ atmosphere, cells were collected and lysed in lysis buffer [10 mM Tris-HCl (pH 8.2), 1% NP40, 1 mM EDTA, 0.1% BSA, 150 mM NaCl] containing 1/50 (vol/vol) of protease inhibitor cocktail (Calbiochem). Western blot assay for signaling-related proteins was carried out as described [33]. Immunoreactive bands were quantified using the image analysis tool ImageJ.

### Flow cytometry analysis

Cells were seeded at the density of 4 × 10^5^ in a 75 cm^2^ tissue culture flask in RPMI 1640 medium supplemented with 10% FBS at 5% CO_2_ atmosphere and incubated with the indicated doses of celecoxib. Untreated cells were used as a control. The DMSO (vehicle of the drug) concentration was maintained at 0.02% in all flasks. Following a 24 h incubation at 37 °C in a 5% CO_2_ atmosphere, cells were collected and cell surface stained as described [34]. Stained cells were analyzed with a flow cytometer (BD FACSAria II, BD Bioacience). Data were analyzed using Kaluza Flow Cytometry Analysis v1.3 software (Beckman Coulter).

### Statistical analysis

Averages, standard deviations (SD), and unpaired *t* test were calculated using MS-Excel. Data are shown as mean ± SD of the results obtained in at least three independent experiments. Statistical analysis was performed with the Stata Statistical Software, Release 13 (StataCorp LP, College Station, TX). Correlation of COX-2 and PD-L1 expression was analyzed by Spearman’s rank correlation coefficient. Difference in the expression of COX-2 in according to PD-L1 groups was analyzed using the Kruskal–Wallis rank test. Correlation of PD-L1 expression with pathological features of the tumor samples was analyzed by Fisher exact test. Differences in the expression levels of COX-2 and pathological features of tumors was analyzed using the Mann–Whitney U-test. Difference in COX-2 or PD-L1 expression in primary and not matched metastatic lesion groups were analyzed using the Mann–Whitney U test. *P* < 0.05 was considered to be statistically significant. All tests used were two-tailed.

## Results

### Tumor specimens

Forty-four samples obtained from patients who underwent surgical resection of their primary melanoma tumors at our institution were analyzed. Table 1 summarizes the tumor pathologic characteristics. Tumor thickness ranged from 0.5 to 10 mm (mean 2.92 ± 2.25). Ulceration was present in 29 (65.9%) of the 44 primary melanoma tumors analyzed. Six (13.6%), 14 (31.8%), 11 (25.0%) and 13 (29.6%) of the 44 primary melanoma tumors were T1, T2, T3 and T4, respectively. BRAF (V600E and V600K) and NRAS (Q61R and Q61L) mutations were detected in 65.9 and 6.8%, respectively, of the primary melanoma tumor biopsies. No mutations in *BRAF* and *NRAS* were detected in the remaining 27.3% of the tumors (Additional file 1: Table S1). In addition, forty-five samples obtained from not matched metastatic lymph node biopsies were analyzed. BRAF (V600E and V600K) and NRAS (Q61R and Q61L) mutations were detected in 57.8 and 8.9%, respectively, of the metastatic melanoma tumor biopsies. No mutations in *BRAF* and *NRAS* were detected in the remaining 33.3% of the tumors (Additional file 1: Table S2).

**Table 1:** Tumor pathologic characteristics of primary melanomas

	#	(%)
Tumor thickness		
Range	0.50–10.00 mm	
Mean	2.92 ± 2.25 mm	
Ulceration		
Absent	15	(34.1)
Present	29	(65.9)
Tumor size		
pT1	6	(13.6)
pT2	14	(31.8)
pT3	11	(25.0)
pT4	13	(29.6)

### PD-L1 expression in primary and not matched metastatic melanoma tissues and its correlation with the pathological features of tumors analyzed

**Fig. 1:**
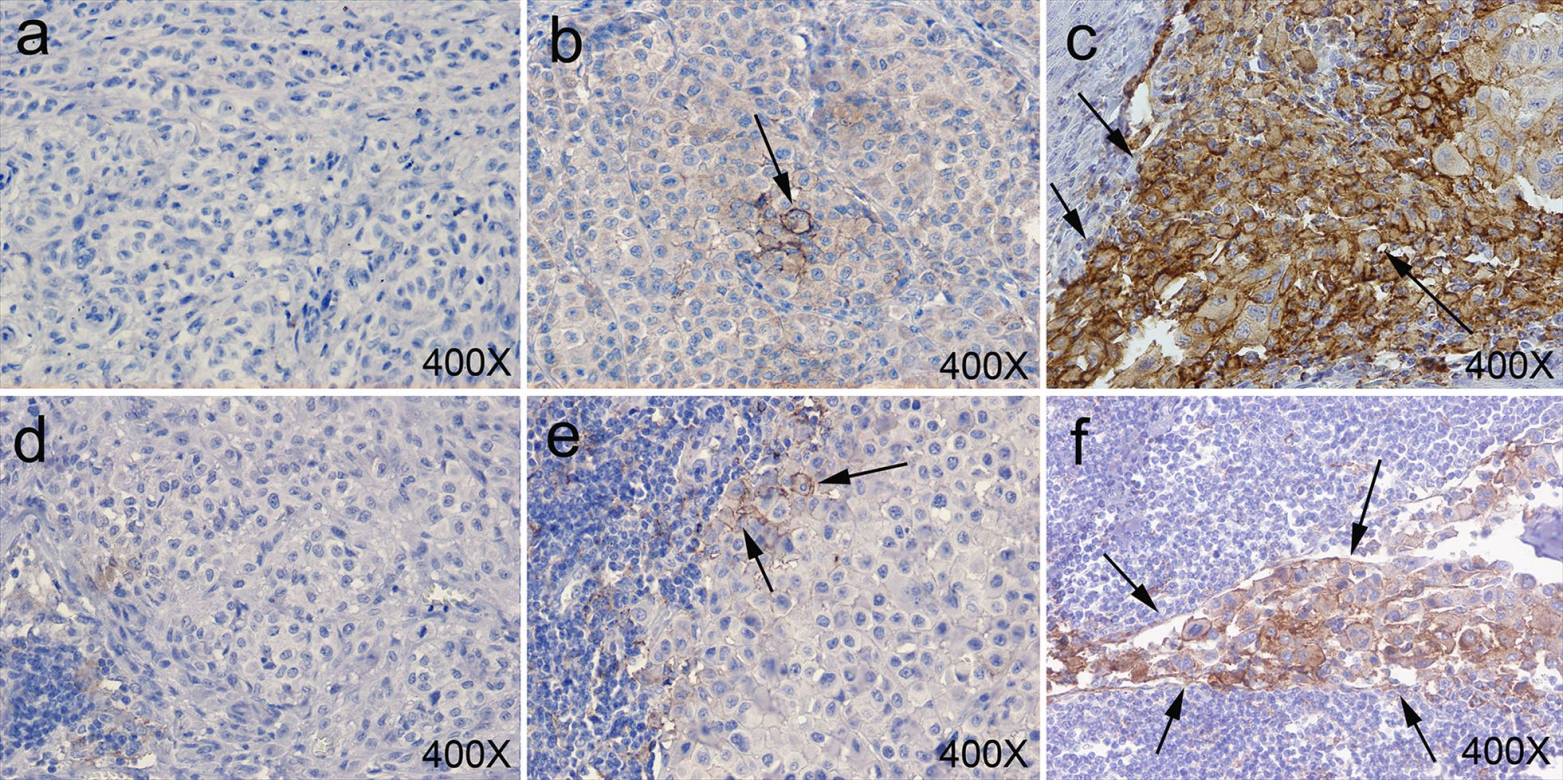
Representative IHC staining patterns with PD-L1-specific mAb (clone SP-142) of FFPE primary (**a**, **b**, **c**) and metastatic lymph node (**d**, **e**, **f**) from a total of 44 primary and 45 not matched metastatic melanoma tumors. PD-L1 expression was reviewed and enumerated independently and blindly by two experienced pathologists (GB and AA). PD-L1 expression was scored as negative (0+) (**a**, **d**), light positive (1+) (**b**, **e**) and positive (2+) (**c**, **f**) when the PD-L1 score in an entire lesion was 0, 1–5, and >5% respectively. Magnification is indicated. *Arrows* indicate examples of PD-L1 positive melanoma cells

PD-L1 positive cancer cells showed a membrane immune reactivity (Fig. 1). PD-L1 by cancer cells was expressed in 55/89 cases (61.8%) of all tumor analyzed. Melanoma cells expressed PD-L1 in 32 (72.7%) and 23 (51.1%) of primary melanoma and not matched metastatic lesions, respectively. In the primary lesions the staining was scored as negative, light positive and positive in 12 (27.3%), 22 (50.0%) and 10 (22.7%) of the 44 lesions, respectively. In the metastatic lesions the staining was scored as negative, light positive and positive in 22 (48.9%), 7 (15.5%) and 16 (35.6%) of the 45 lesions, respectively. Statistical analysis demonstrated a significantly lower number of PD-L1 negative lesions in primary tumors as compared to not matched metastatic lesions (Mann–Whitney U test*, P* = 0.002).

No correlation was identified between PD-L1 expression on melanoma cells and the presence of ulceration and the T stage of the primary melanoma lesions. In addition no correlation was identified between PD-L1 expression on melanoma cells and the BRAF/NRAS genotype of the lesions analyzed.

### COX-2 expression in primary and not matched metastatic melanoma tissues and its correlation with the pathological features of tumors analyzed

**Fig. 2:**
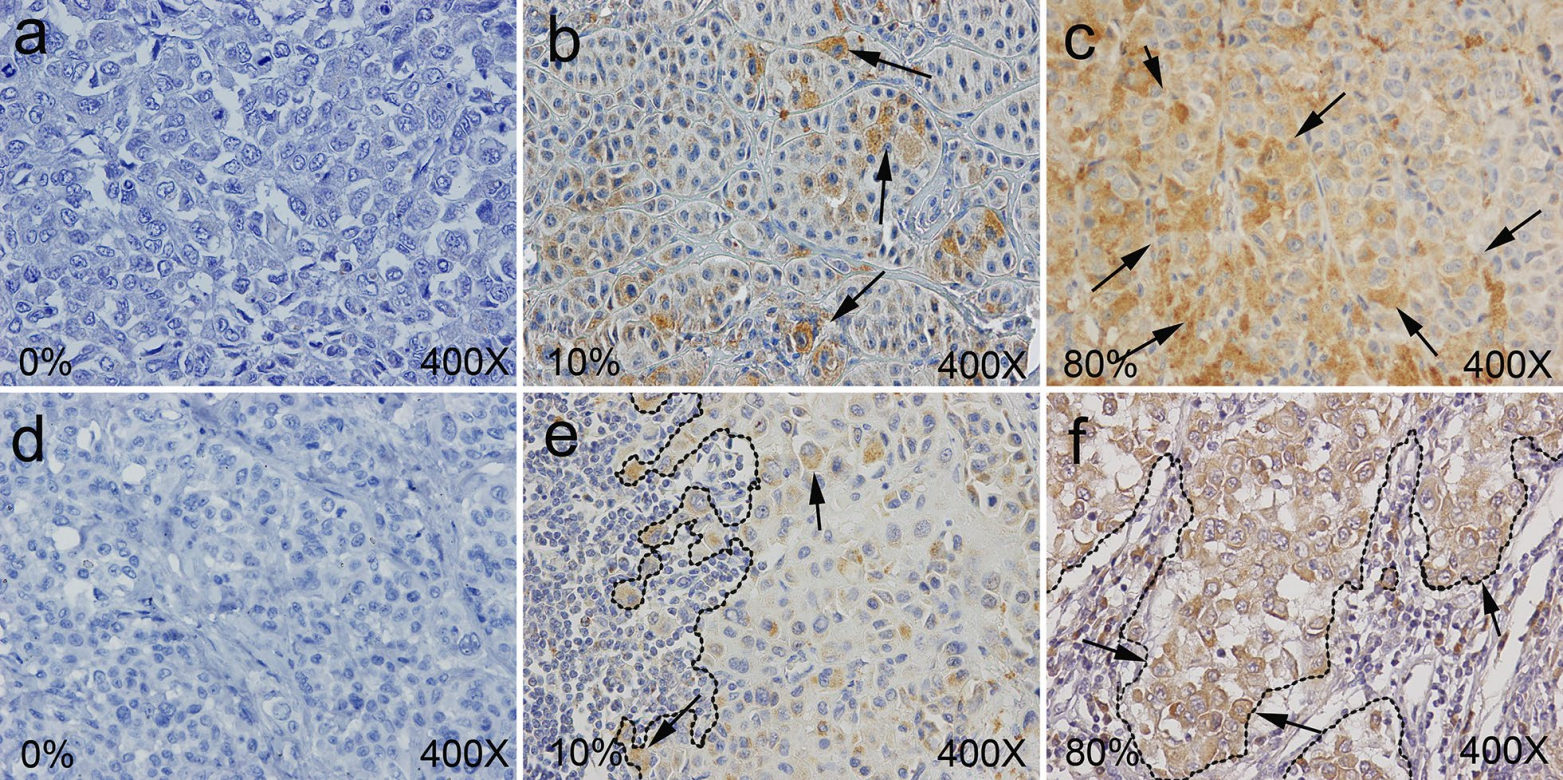
Representative IHC staining patterns with COX-2-specific mAb (clone CX-294) of FFPE primary (**a**, **b**, **c**) and metastatic lymph node (**d**, **e**, **f**) from a total of 44 primary and 45 not matched metastatic melanoma tumors. COX-2 expression was reviewed and enumerated independently and blindly by two experienced pathologists (GB and AA). COX-2 expression was scored as variations in the percentage of stained cells at 10% intervals. Staining was graded as a semi-quantitative variable. Results ranged from 0 to 80% of positive melanoma cells. Percentage of COX-2 positive cells, tumor margin (*dotted line*) and magnification are indicated. *Arrows* indicate examples of COX-2 positive melanoma cells

COX-2-positive cells showed brownish granules in the cytoplasm (Fig. 2). COX-2 by cancer cells was expressed in 82/89 (92.1%) cases of all tumor analyzed. COX-2 expression was negative on melanoma cells in 2 out of 44 primary melanomas and 5 out of 45 metastatic lesions. In both primary and not matched metastatic lesions COX-2 expression in melanoma cells ranged from 0 to 80%. The mean expression of COX-2 in primary tumors was 43.8 ± 23.4% while it was 38.0 ± 23.2% in the metastatic lesions. No significant difference was found in COX-2 expression between primary and not matched metastatic melanoma lesions. No correlation was identified between COX-2 expression in melanoma cells and the presence of ulceration or the T stage of the primary melanoma lesions. In addition no correlation was identified between COX-2 expression in melanoma cells and the BRAF/NRAS genotype of the lesions analyzed.

### PD-L1 and COX-2 correlation in primary and metastatic melanoma tissues

**Fig. 3:**
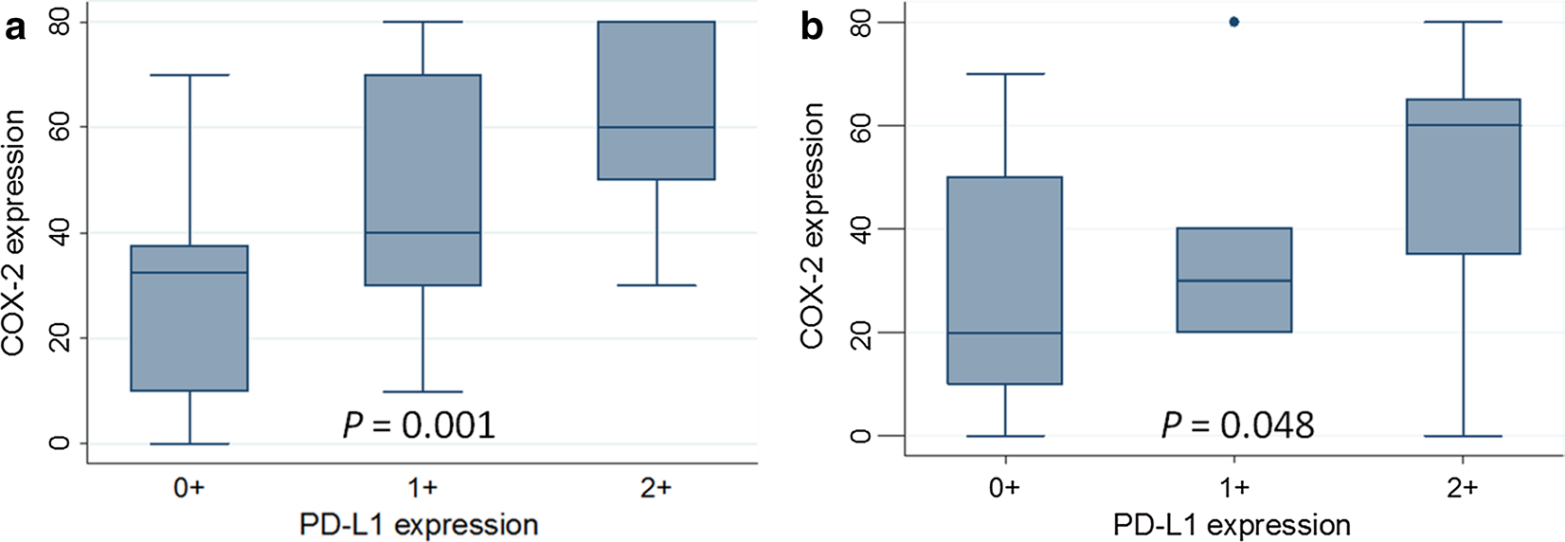
Correlation between PD-L1 and COX-2 expression in primary (**a**) and metastatic lymph node (**b**) of melanoma tumors. COX-2 expression, graded as a semi-quantitative variable, was correlated with PD-L1 expression scored as negative (0+), light positive (1+) and positive (2+) when the PD-L1 score in an entire lesion was 0, 1–5, and >5% respectively. Difference in the expression of COX-2 in according to PD-L1 groups was analyzed using the Kruskal–Wallis rank test. On *each box*, the central mark is the median, the edges of the box are the 25th and 75th percentiles, the *whiskers* extend to the most extreme data points not considered outliers, and outliers are plotted individually. *P* value is indicated

PD-L1 expression significantly correlated with COX-2 expression in the melanoma lesions analyzed (Kruskal–Wallis rank test, *P* = 0.0008). Specifically, a significant correlation between COX-2 expression and PD-L1 was found in both primary (Kruskal–Wallis rank test, *P* = 0.001) and not matched metastatic (Kruskal–Wallis rank test, *P* = 0.048) lesions. PD-L1 was expressed in a greater extent in melanoma tissues which express a higher level of COX-2 (Spearman’s rank correlation coefficient, spearman rho: +0.4376, *P* = 0.0001). The mean expression of COX-2 was 27.9 ± 20.0%, 45.2 ± 22.2% and 60.0 ± 18.8% in primary melanoma lesions which were scored for PD-L1 as negative, light positive and positive, respectively (Fig. 3a). The mean expression of COX-2 was 30.9 ± 23.0%, 35.7 ± 21.4% and 48.7 ± 21.2% in metastatic melanoma lesions which were scored for PD-L1 as negative, light positive and positive, respectively (Fig. 3b).

### PD-L1 and COX-2 co-localize in primary and not matched metastatic melanoma tissues

**Fig. 4:**
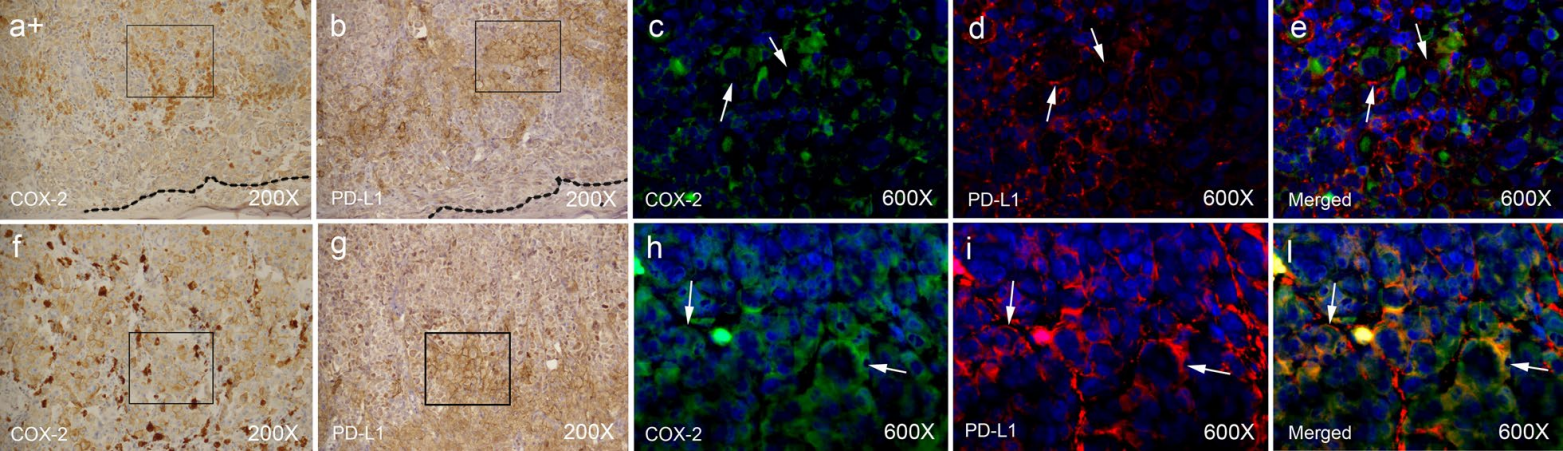
Representative IHC (**a**, **b**, **f**, **g**) and matched immunofluorescent staining (**c**, **d**, **e**, **h**, **i**, **l**) patterns of FFPE primary (**a**, **b**, **c**, **d**, **e**) and not matched metastatic lymph node (**f**, **g**, **h**, **i**, **l**) of melanoma tumors with COX-2-specific mAb (clone CX-294) (**a**, **c**, **f**, **h**), PD-L1-specific mAb (clone SP-142) (**b**, **d**, **g**, **i**) and both COX-2- (clone CX-294) and PD-L1-specific mAbs (clone SP-142) (**e**, **l**). Immunofluorescent staining matches to squared field of IHC staining. COX-2 was detected by goat anti-mouse IgG dylight 488 (*green*). Immunofluorescent staining of PD-L1 was detected by goat anti-rabbit IgG dylight 594 (*red*). Nuclei were stained by DAPI (*blue*). Negative controls are provided in Additional file 1: Figure S1. *Arrows* indicate examples of positive cells. Tumor margin (*dotted line*) and magnification are indicated

We then investigated whether single melanoma cells expressing PD-L1 were also co-expressing COX-2. A double-immunofluorescence staining utilizing 12 representative lesions, six each from primary and not matched metastatic melanomas, was performed. Representative staining patterns of primary and metastatic melanoma lesions with both PD-L1- and COX-2-specific mAbs are shown in Fig. 4. In all 12 melanoma tissue sections analyzed from both primary and not matched metastatic lesions the majority of cancer cells which express PD-L1 also co-expressed COX-2 while most of the cells which did not express PD-L1 did not express COX-2.

### COX-2 inhibition down-regulates PD-L1 expression in both BRAF and NRAS mutant melanoma cell lines

**Fig. 5:**
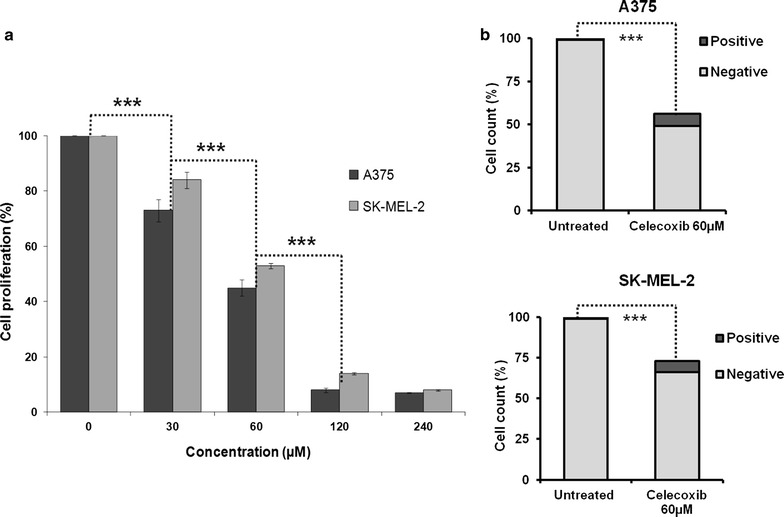
Effect of celecoxib on the in vitro proliferation of BRAF^V600E^ and NRAS^Q61R^ melanoma cell lines. **a** BRAF^V600E^ A375 and NRAS^Q61R^ SK-MEL-2 melanoma cells were seeded at the density of 3 × 10^3^ per well in a 96-well plate and incubated with the indicated concentrations of celecoxib. Untreated cells were used as a control. DMSO (vehicle of celecoxib) concentration was maintained at 0.02% in all wells. Following a 24 h incubation at 37 °C in a 5% CO_2_ atmosphere, growth inhibition was determined by MTT assay. Data are expressed as mean percent of proliferation ± SD of treated cells as compared to untreated control cells. Mean percent of proliferation and SD were calculated from three independent experiments performed in triplicate. Difference between doses of celecoxib was calculated using unpaired *t*-test. *** indicate *P* < 0.001. **b** BRAF^V600E^ A375 and NRAS^Q61R^ SK-MEL-2 melanoma cells were seeded at the density of 4 × 10^5^ per well in a 75 cm^2^ tissue culture flask and incubated with celecoxib (60 μM). Untreated cells were used as a control. DMSO (vehicle of celecoxib) concentration was maintained at 0.02% in all wells. Following a 24 h incubation at 37 °C in a 5% CO_2_ atmosphere, viability of cells was determined by trypan blue assay. Data are expressed as mean percentage of viable (*negative*) and death cells (*positive*) of treated cells as compared to untreated control cells. *** indicate *P* < 0.001

**Fig. 6:**
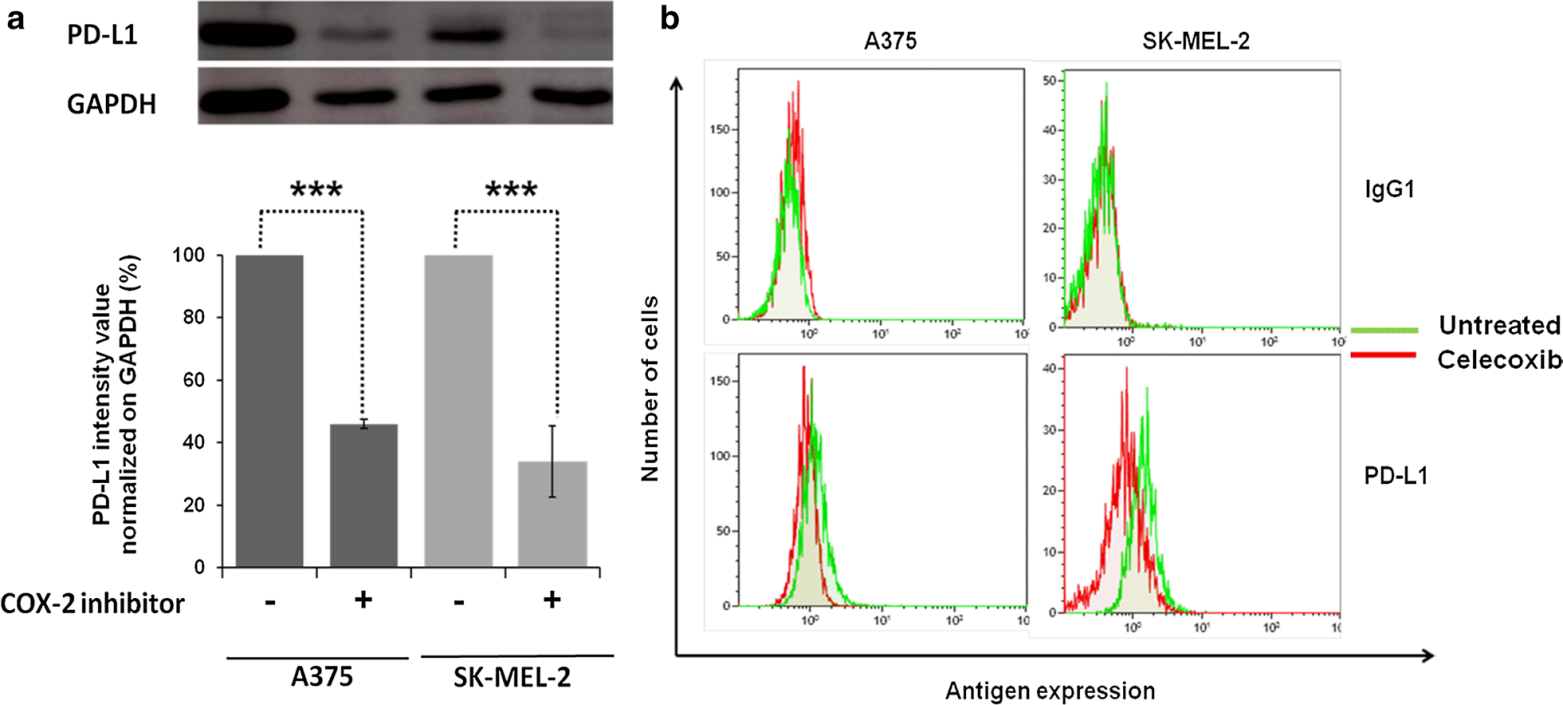
Down-regulation of PD-L1 by celecoxib in BRAF^V600E^ and NRAS^Q61R^ melanoma cell lines. BRAF^V600E^ A375 and NRAS^Q61R^ SK-MEL-2 melanoma cells were seeded at the density of 4 × 10^5^ per well in a 75 cm^2^ tissue culture flask and incubated with celecoxib (60 μM). Untreated cells were used as a control. DMSO (vehicle of celecoxib) concentration was maintained at 0.02% in all wells. **a** Following a 24 h incubation at 37 °C in a 5% CO_2_ atmosphere, cells were harvested and lysed. Cell lysates were analyzed by western blot with the indicated mAbs. PD-L1 was detected using the PD-L1-specific mAb (clone SP-142). GAPDH was used as a loading control. Representative results are shown (*upper panel*). The levels of PD-L1 normalized to GAPDH are plotted and expressed as mean ± SD of the results obtained in three independent experiments (*bottom panel*). *** indicate *P* < 0.001. **b** Following a 24 h incubation at 37 °C in a 5% CO_2_ atmosphere, cells were harvested and cell surface stained with the PE-conjugated PD-L1-specific mouse mAb [clone MIH1 (RUO)]. PE-conjugated mouse IgG1 was used as a specificity control. Representative results are shown

We lastly investigated whether PD-L1 expression was modulated by COX-2 activity by testing PD-L1 expression following inhibition of COX-2. The human BRAF^V600E^ A375 and NRAS^Q61R^ SK-MEL-2 melanoma cell lines were used as an in vitro model. A375 and SK-MEL-2 were treated with the selective COX-2 inhibitor celecoxib at different doses. As shown in Fig. 5a, following a 24 h incubation, celecoxib inhibited the growth of both A375 and SK-MEL-2 melanoma cell lines in a dose-dependent manner. The dose of 60 μM inhibited the growth of A375 and SK-MEL-2 melanoma cells of about 50%. This effect was associated with induction of cell death and prevention of cell proliferation in both A375 and SK-MEL-2 cells (Fig. 5b). The induction of cell death was more marked in A375 cell line than in SK-MEL-2 cells. Based on these findings, we treated A375 and SK-MEL-2 cells with 60 μM of COX-2 inhibitor and analyzed the expression of PD-L1. Both western blot (unpaired *t* test, *P* < 0.001) and flow cytometry (unpaired *t* test, *P* < 0.02) analyses demonstrated that treatment with celecoxib significantly down-regulated the total protein level and surface expression of PD-L1 in both A375 and SK-MEL-2 melanoma cells as compared to untreated cells (Fig. 6).

## Discussion

Targeting the immune checkpoint molecules CTLA-4, PD-1 and PD-L1 is completely revolutionizing the treatment of advanced melanoma. Several clinical trials have convincingly shown that immune checkpoint inhibitors increase the overall survival of metastatic melanoma patients with or without BRAF/NRAS mutations [2–6]. However the response rate of these novel immunotherapeutic approaches is about 40–60%. A growing body of evidence indicates that a major obstacle to the success of immunotherapy is represented by the development of escape mechanisms utilized by tumor cells to avoid recognition and destruction by the host’s immune system. The PD-1/PD-L1 axis represents one of the major mechanisms of tumor immune escape since expression of PD-L1 on cancer cells through the interaction with PD-1 on T cells inhibits the recognition and destruction of cancer cells by the host immune response.

So far PD-L1 expression by cancer cells correlates with melanoma progression. Furthermore PD-L1 expression by cancer cells is currently investigated as a potential biomarker in order to predict clinical responses to anti-PD-1 and -PD-L1 based immunotherapy in several malignancies [35]. In addition a higher expression of PD-L1 in tumors appears to predict for a higher clinical response rate in patients treated with anti-PD-1/PD-L1 based immunotherapy as compared to patients which carry tumors that did not express PD-L1. Nevertheless, some patients not expressing PD-L1 in their tumors also benefit from PD-1/PD-L1 based immunotherapy [36]. These findings might reflect the dynamic expression of PD-1/PD-L1 by both cancer cells and immune cells. Indeed PD-1/PD-L1 expression can be induced on immune cells and cancer cells by the presence of soluble factors or cytokines present in tumor microenvironment. The latter are induced by the interaction of cancer cells with host immune cells during treatment with anti-PD-1/PD-L1 mAbs. In addition PD-1/PD-L1 expression can be induced by pro-tumorigenic pathways which are activated in cancer cells during cancer progression [14]. In this study we showed a relationship between COX-2 and PD-L1 expression in melanoma cells. COX-2 is an important key survival molecule, currently investigated as a molecular marker and a potential tumor therapeutic target. In fact, the prophylactic use of COX-2 inhibitors such as acetylsalicylic acid has been shown to decrease the incidence of several cancers. In particular, its synergistic activity with other anti-tumoral treatments, such as chemotherapy and radiotherapy [37–39] or with other molecular targets related to tumor growth and proliferation, including VEGFR or aromatase inhibitors [40–42] has been often highlighted. In their recent published work Markosyan et al. [22] describe a cause effect relationship between COX-2 and PD-L1 expression in breast cancer cells. In addition Prima et al. describe that COX-2 and the PGE2 pathway regulates PD-L1 expression in tumor-associated macrophages and myeloid-derived suppressor cells [43]. Our data in melanoma cells are in line with these findings since COX-2 is significantly associated with PD-L1 in melanoma tumors and its inhibition by the COX-2 inhibitor celecoxib down-regulates the expression of PD-L1 in vitro. There is no clear evidence in the literature of the mechanisms which might underlie the effect on PD-L1 by COX-2. Some works reported a relationship between COX-2 and activation of AKT/STAT3 or NF-κB pathways [44, 45] which are known to regulate PD-L1 expression [46–49]. However further studies are warranted to determine the underlying mechanisms. Future studies addressing the molecular signals that regulate PD-L1 surface expression by COX-2 in cancer cells might shed light on developing novel therapeutic agents to modulate cancer cell immune responses.

COX-2 isoform is shown to be up-regulated during both inflammation and cancer [16, 19]. In melanoma COX-2 is shown to be expressed in both primary and metastatic lesions and its expression correlates with pathological features of primary tumors and patient’s prognosis [28, 50, 51]. Our data are in line with previous reports since COX-2 by cancer cells in melanoma tumors is found to be highly expressed in both primary and not matched metastatic melanoma lesions although no significant correlation between COX-2 and the pathological features of tumor analyzed was found. These findings may reflect the low number of lesions analyzed as well as the selection of the tumors.

A body of evidence indicates a role for COX-2 in the development/modulation of different steps of cancer progression and several mechanisms may underlie this pro-tumorigenic activity of COX-2 including (i) production of reactive oxygen species responsible for DNA damage, (ii) aberrant activation of intracellular pathways such as MAPK and the PI3 K/AKT pathways, (iii) activation of STAT3, (iv) induction of Bcl-2 family members and (v) production of growth factors including epidermal growth factor (EGF) and fibroblast growth factor (FGF) [16]. These in turn favor the increase rate of mutations, the proliferation, pro-survival signals and initiated tumor cells by determining an imbalance between cell proliferation and cell death stimuli. Our data demonstrated that COX-2 is involved in the proliferation of melanoma cells since treatment of melanoma cells with or without BRAF/NRAS mutations with the COX-2 inhibitor celecoxib inhibits their proliferation and induces cell death. This effect are in line with previous work [21, 52, 53], providing COX-2 inhibition as an additional valid option in order to inhibit melanoma cell progression. The molecular mechanisms underlying this anti-tumor activity of celecoxib in melanoma cells was not fully investigated in the present study. In A375 cell line inhibition of cell proliferation and/or induction of cell death by celecoxib was associated with down-regulation of Bcl-2. These data are line with previous works which have reported that Bcl-2 down-regulation by COX-2 inhibition is mediated by NF-kB [45]. In contrast in SK-MEL-2 cell line treatment with celecoxib did not change the expression of Bcl-2 which results to be not expressed (Additional file 1: Figure S2). Further studies are needed to clarify the molecules which are involved in inducing cell death and/or preventing cell proliferation following treatment with COX-2 inhibitor in this cell line.

Lastly COX-2 is shown to contribute to immune evasion and resistance to cancer immunotherapy in several cancer types including breast, lung, colon cancer and melanoma [22, 52, 54–57]. COX-2 has been involved in suppressing the activity of immune cells including dendritic cells, natural killer and T cells as well as in promoting type-2 immunity which causes tumor immune evasion. In the present work the activity of COX-2 and its inhibition on immune cell activation/proliferation and/or on the interaction between immune cells and melanoma cells was not investigated. However the novel information we show is that in melanoma cells COX-2 activity causes tumor immune escape by modulating PD-L1 expression. Indeed COX-2 is found to be co-expressed to PD-L1 in both primary and not matched metastatic lesions and inhibition of COX-2 by celecoxib down-regulates PD-L1 in melanoma cells. The latter plays a major role in inhibiting the host immune response.

## Conclusions

Over the past years the possibility to combine different therapies, such as biological therapy and immunotherapy, has contributed to completely change the setting of treatment plans [58]. The limited duration of the response obtained with BRAF and MEK inhibition in BRAF^V600^ melanoma and the impressive durability but the relatively low response rate obtained with the block of the immune checkpoint molecules proved to be surprising. Several clinical trials are examining the combinations of different immune checkpoint inhibitors as well as the combination of immune checkpoint inhibitors with the small molecule inhibitors [59–62]. In this view our data shown that COX-2 expression correlates with and modulates PD-L1 expression in melanoma cells. Besides modulating PD-L1 expression, COX-2 is also shown to be involved in melanoma progression since its inhibition induces cell death and prevents cell proliferation. These findings have clinical relevance since they provide a rationale to implement novel clinical trials to test COX-2 inhibition as a potential treatment to prevent melanoma progression and immune evasion as well as to enhance the anti-tumor activity of PD-1/PD-L1 based immunotherapy for the treatment of melanoma patients with or without BRAF/NRAS mutations.

## Additional file

**Additional file 1.** Additional figures and tables.

## Abbreviations

AJCC: American Joint Committee on Cancer
ATCC: American Type Culture Collection
COX: cyclooxygenase
CTL: cytotoxic T lymphocytes
CTLA-4: cytotoxic T lymphocyte antigen 4
DAB: 3,3′-diaminobenzidine
DAPI: 4′,6′-diamidino-2-phenylindole
DMSO: dimethyl sulfoxide
FBS: fetal bovine serum
FFPE: formalin-fixed paraffin embedded
h: hour
H&E: hematoxylin and eosin
HRP: horseradish peroxidase
IHC: immunohistochemical
IRB: institutional review board
MAPK: MAP kinase
mAb: monoclonal antibody
min: minute
MTT: 3-(4,5-dimethylthiazol-2-yl)-2,5-diphenyltetrazoliumbromide
NSAIDs: non-steroidal anti-inflammatory drugs
PD-1: programmed death receptor 1
PE: R-phycoerythrin
PD-L1: programmed death receptor ligand 1
RT: room temperature
SD: standard deviations
