# Supplementary material for: COX-2 expression positively correlates with PD-L1 expression in human melanoma cells
Source: J Transl Med. 2017 Feb 23;15:46. doi: 10.1186/s12967-017-1150-7 (PMC5324267; doi:10.1186/s12967-017-1150-7)
Supplement: Supplementary file 1 — Additional file 1. Additional figures and tables. [file 12967_2017_1150_MOESM1_ESM.docx]

**Supplementary Table S1.** Characterization of BRAF and NRAS genotypes in tumor biopsies obtained from primary melanomas.


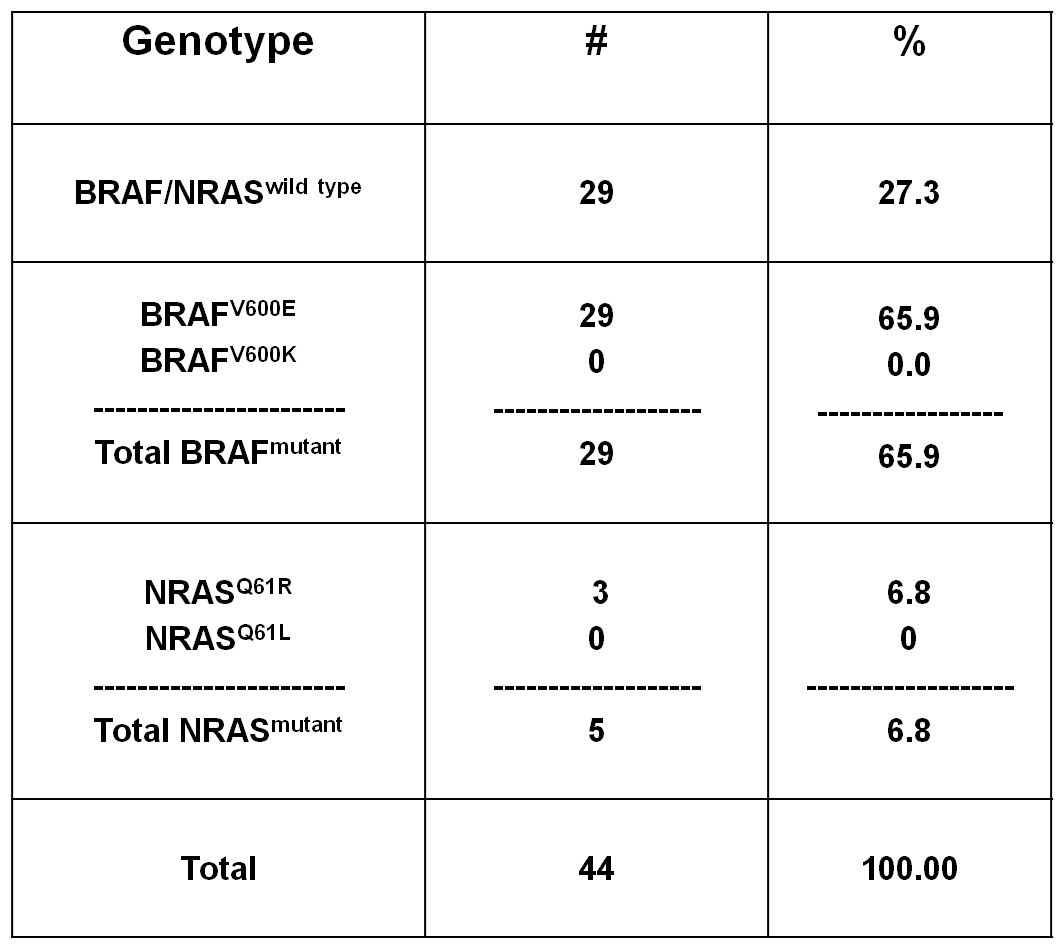


**Supplementary Table S2.** Characterization of BRAF and NRAS genotypes in tumor biopsies obtained from metastatic melanoma lymph nodes.


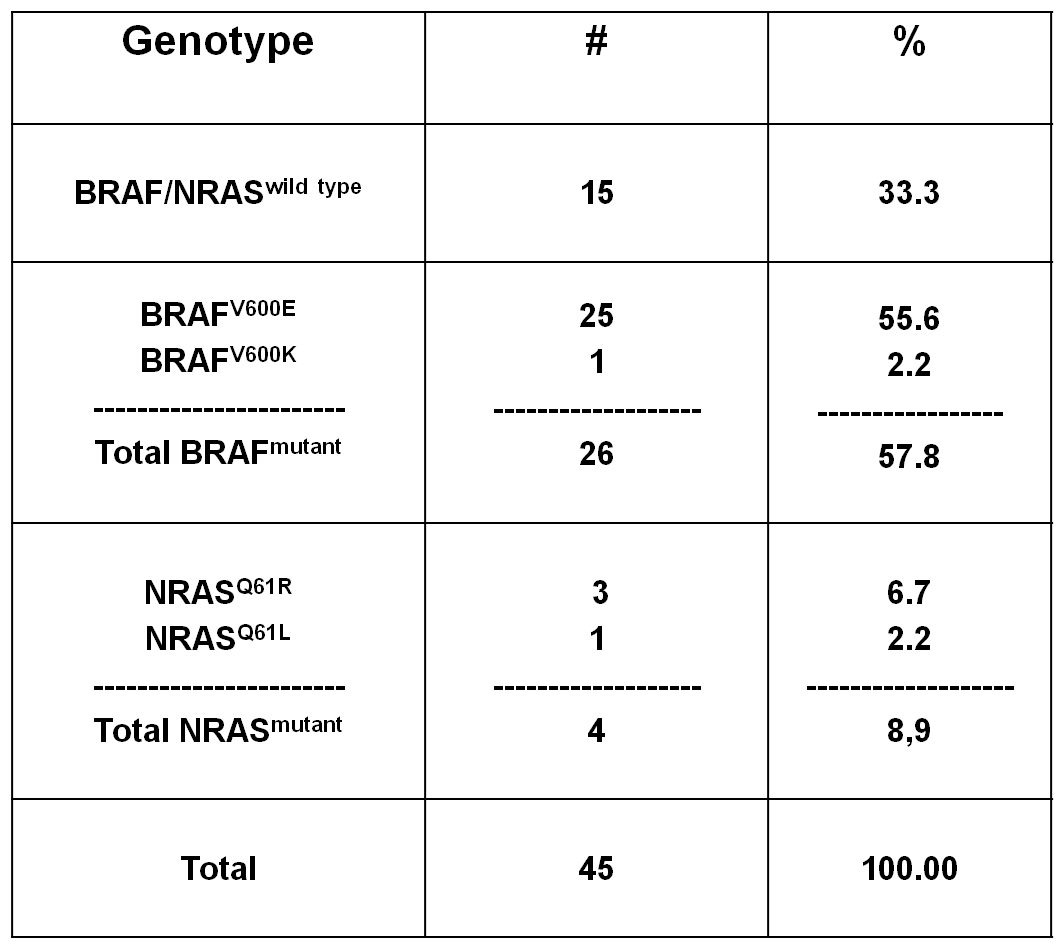


**Supplementary Data**

**Supplementary Figure S1. Negative controls of representative immunofluorescent staining patterns of FFPE primary (left panel) and not matched metastatic lymph node (right panel) of melanoma tumors.** A cocktail of goat anti-mouse IgG dylight 488 (green) and goat anti-rabbit IgG dylight 594 (red) were used as a negative controls. Nuclei were stained by DAPI (blue). Arrows indicate examples of melanoma cells. Magnification is indicated.


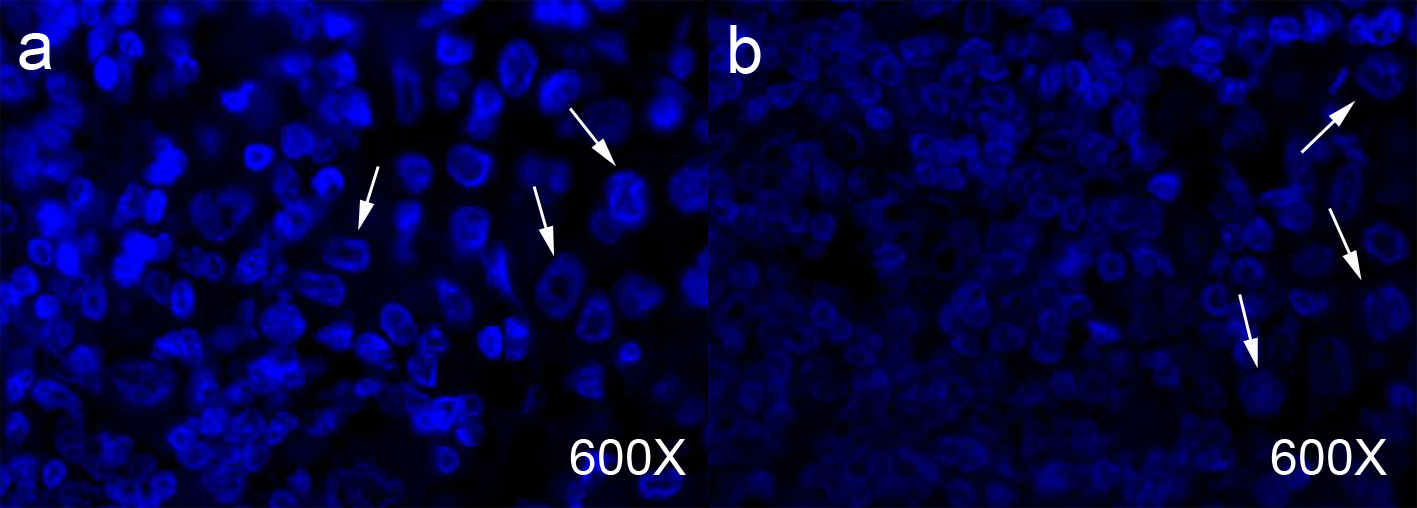


**Supplementary Figure S2.** BRAF^V600E^ A375 and NRAS^Q61R^ SK-MEL-2 melanoma cells were seeded at the density of 4 x 10^5^ per well in a 75 cm^2^ tissue culture flask and incubated with celecoxib (60 uM). Untreated cells were used as a control. DMSO (vehicle of vemurafenib) concentration was maintained at 0.02% in all wells. Following a 24 h incubation at 37°C in a 5% CO_2_ atmosphere, cells were harvested and lysed. Cell lysates were analyzed by western blot with the Bcl-2-specific mAb. β-actin was used as a loading control. Representative results are shown (upper panel). The levels of Bcl-2 normalized to β-actin are plotted and expressed as mean ± SD of the results obtained in two independent experiments (bottom panel). *** indicate *P*= 0.03
